# Additive manufacturing of metallic glass from powder in space

**DOI:** 10.1038/s41526-023-00327-7

**Published:** 2023-10-06

**Authors:** Christian Neumann, Johannes Thore, Mélanie Clozel, Jens Günster, Janka Wilbig, Andreas Meyer

**Affiliations:** 1 Institut für Materialphysik im Weltraum, Deutsches Zentrum für Luft- und Raumfahrt (DLR), Linder Höhe, 51170 Köln, Germany; 2 Bundesanstalt für Materialforschung und –prüfung (BAM), Unter den Eichen 87, 12205 Berlin, Germany

**Keywords:** Materials science, Aerospace engineering

## Abstract

Additive manufacturing of metals – and in particular building with laser-based powder bed fusion – is highly flexible and allows high-resolution features and feedstock savings. Meanwhile, though space stations in low Earth orbit are established, a set of visits to the Moon have been performed, and humankind can send out rovers to explore Venus and Mars, none of these milestone missions is equipped with technology to manufacture functional metallic parts or tools in space. In order to advance space exploration to long-term missions beyond low Earth orbit, it will be crucial to develop and employ technology for in-space manufacturing (ISM) and in-situ resource utilisation (ISRU). To use the advantages of laser-based powder bed fusion in these endeavours, the challenge of powder handling in microgravity must be met. Here we present a device capable of building parts using metallic powders in microgravity. This was proven on several sounding rocket flights, on which occasions Zr-based metallic glass parts produced by additive manufacturing in space were built. The findings of this work demonstrate that building parts using powder feedstock, which is more compact to transport into space than wire, is possible in microgravity environments. This thus significantly advances ISRU and ISM and paves the way for future tests in prolonged microgravity settings.

## Introduction

Additive manufacturing (AM) technologies appear to be a tremendous opportunity to meet spaceflight requirements, because they contribute to saving material, reducing mass to transport, and reducing production time. Furthermore, they allow for usage of materials recycled or directly collected on-site, so-called in-situ resource utilisation (ISRU). In the previous decade, NASA and Made In Space, Inc., brought an FFF (Fused Filament Fabrication) printer to the International Space Station, proving the feasibility of polymer AM in microgravity^1,2^.

Laser-based Powder Bed Fusion (PBF-LB) is one of the most versatile AM processes in terms of possible geometries and scalable process parameters, and is adaptable to a wide range of materials^3–5^ such as metals^6–9^, ceramics and glasses^10–12^, and polymers^13,14^. It relies on a flowable powder being spread over a build-platform and melted by a laser in a chosen 2D geometry. The platform is subsequently lowered and the next layer of powder is spread above the previous one. Over the recent years this technology has matured and become a reliable alternative for manufacturing structural parts^15^, and for manufacturing parts with complex geometry or from materials difficult to process in traditional ways^16^.

Metallic glasses are a relatively recent class of materials, dating back to the early 1960s. Depending on composition, they possess attractive properties^17,18^ such as excellent corrosion resistance, good mechanical properties, low friction coefficient. NASA’s BMGG project (bulk metallic glass gears, https://www.nasa.gov/directorates/spacetech/game changing development/projects/BMGG, last consulted 18.01.2023) aims to develop gearboxes made from BMG which would require neither lubrication nor heating, an example of their potential. The greatest impediment to the use of BMGs as structural parts and tools, despite their advantageous properties and the huge improvements made over the last decades, is the size of manufactured parts. This is usually limited to a few millimetres to centimetres in thickness due to falling cooling rates and increasing crystallisation when increasing thickness during casting^19^. Only recently have these materials been used within the context of AM. By building layer by glassy layer, this process was revealed to allow circumventing of these size limitations^7,9,20^ and it is possible to form an amorphous part thicker than that attained by casting.

Therefore, both BMGs and PBF-LB have characteristics attractive to space applications. However, their combination in this domain has seen little research. To combine these two topics – to manufacture parts from bulk metallic glass in a powder-based process independent from gravitational environment – there are several options for experimenting in microgravity conditions, with increasing microgravity times offered: drop towers^21–23^ (microgravity time typically <10 s, residual acceleration around 10^−4^ g), parabolic flights^21,24–28^ (~20 s per parabola, ~10^−3^ g), sounding rockets^21,29–33^ (~400 s, 10^−4^ to 10^−6^ g), and orbital platforms^34–37^ (hours to months, 10^−3^ to 10^−5^ g).

Sounding rockets offer a good compromise between availability, cost, and microgravity time, and were therefore chosen as an experimental environment after the system was qualified for microgravity application in parabolic flights. We consider sounding rocket flights to be a necessary transitional phase in the development of an on-orbit AM device which would provide enough microgravity time to produce a functional metal part.

## Methods

### Payload for sounding rocket flight

This work therefore aims to additively manufacture metallic glass parts in microgravity using PBF-LB, that is to say using a powder feedstock. The PBF-LB process was chosen for its flexibility. PBF-LB usually relies on Earth’s gravitational forces to maintain the powder layers in contact with the build-platform. Therefore, handling the powder feedstock is the biggest challenge under reduced or absent gravitational forces. To compensate for this, the gas flow-assisted powder deposition was developed by Zocca et al. ^24,28^ in which a porous build-platform is used in combination with a vacuum pump-driven reduced pressure to create a gas flow through the build-platform, thus stabilising the powder. This powder handling system was tested and improved upon several DLR (German Aerospace Centre) and ESA (European Space Agency) zero-g parabolic flights^24,28^, but so far, laser melting was not conducted under microgravity.

Sounding rocket flights on the other hand provide several minutes of microgravity time which is enough to deposit several layers of powder and laser-melt a geometry. Therefore, based on the apparatus developed by Zocca et al. for parabolic flight, a device capable of handling powder and building a part in microgravity was engineered and manufactured by the DLR’s Institute of Materials Physics in Space to fit onboard a DLR MAPHEUS sounding rocket^30,31^ in order to demonstrate the feasibility of this powder stabilisation concept.

This device had to include significant modifications from the parabolic flight apparatus to become a sounding rocket payload. Among other things, the device needed to be smaller, resist much higher acceleration forces, be capable of remote communication, and be resilient to space vacuum environment.

For hardware meeting these requirements, the MAPHEUS sounding rocket offers microgravity time ~6.5 min with residual acceleration levels as low as 10^−4^ to 10^−6^ g on a suborbital flight over 90 km above ground level, with an apogee of 260 km. MAPHEUS payload support systems by DLR’s Mobile Rocket Base^32,38,39^ supply the scientific payload with a bi-directional data communication interface and a live video downlink, enabling the operators at the setup ground station to make use of telemetry to supervise the fully automated manufacturing process in real-time, and use telecommand to manually intervene in the process if necessary.

### General experimental set-up

The device must fulfil many requirements to function onboard the sounding rocket. There are environmental requirements: the device must fit within certain dimensions, resist the dynamic loads and vibrations arising during rocket launch, be fit for vacuum external conditions, stabilise the powder in microgravity, and include a cooling system independent of air convection. Most components are off-the-shelf and as such are not designed to resist rocket launch accelerations nor to function under vacuum. Therefore, all components had to undergo a work-over to improve mechanical strength and resilience and vacuum resistance. In addition, there are accessibility requirements: it must possess a powder container and build-platform that are still easily accessible once the device is in flight configuration (i.e., physically connected to the rest of the payload and on the launcher), record process data on-board and transmit them to the monitoring user on-ground, and allow the user to interrupt the building process in case of malfunction. Finally, the device must contain its own power source and be capable of building a part without human intervention in a hermetically closed experiment chamber.

**Fig. 1:**
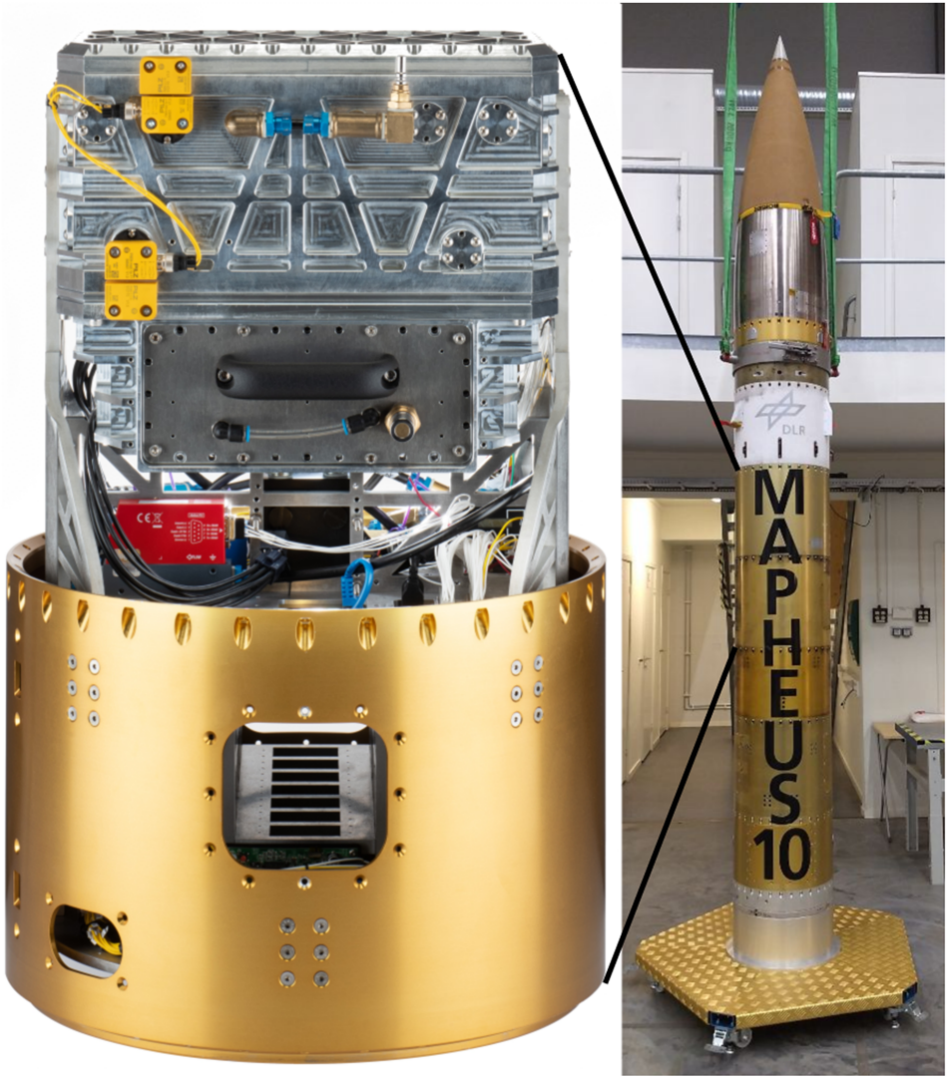
MARS-M rocket payload module. MARS-M (left) and the MAPHEUS-10 rocket payload (right) of which it is part.

The resulting designed and constructed device called MARS-M (Multimaterial Additive manufacturing for Research and Spaceflight, for MAPHEUS) is shown in Fig. 1 (left) and is 700 mm in total length, 438 mm in diameter, at 44 kg net weight plus the outer structure of the rocket payload at 12 kg. It contains a compact and lightweight, fully automated cartesian AM device, including control computing, data acquisition and handling, powder stabilisation, and electrical power supply.

### Moving mechanics

**Fig. 2:**
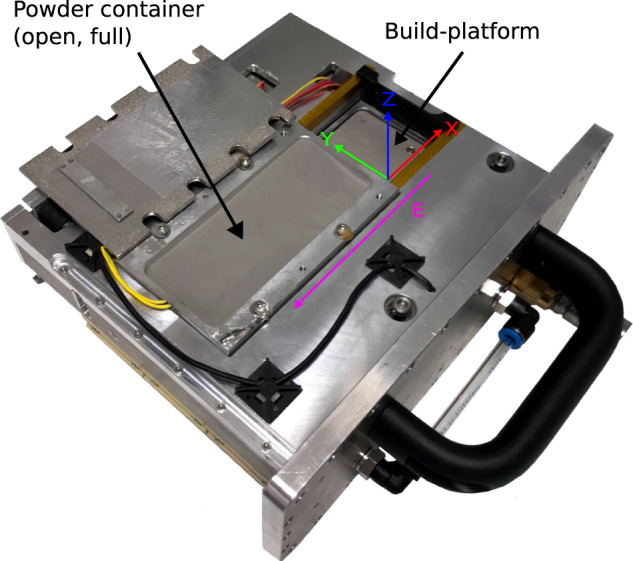
Cartridge holding the build-platform (here, clear of powder) and the powder container. The *X*, *Y*, *Z*, and *E* axes are depicted. The *X* and *Y* axes are in plane with the build-platform. The *Z* axis is perpendicular to the platform. The platform is born under the powder container along the *E* axis to spread each powder layer.

Next to its function of providing the necessary steps to build a part (laser scanning, powder layer application, and build-platform displacement), the basic mechanical set-up must be robust enough to withstand static and dynamic loads during the different phases of the flight. To fulfil both requirements, a cartesian assembly of X- and Y axes is used to move a focused laser spot in a horizontal plane above the build-platform, and a vertical Z axis to lower the build-platform as the part thickness increases (see Fig. 2). An additional axis E is used to apply fresh powder layers. Both X- and Y axes use Ø 8 mm shafts and self-lubricating dry-running polymer bearings as linear carriages to prevent powder particles from sticking to grease and blocking movements. For the shafts, carbon-fibre-reinforced polymer (CRP) and hard-anodised aluminium were used to reduce mass. Each axis is driven by a stepper drive via a timing belt, and limited by optical end stop switches. The Y axis moves the X axis, which in turn moves a socket bearing the fibre-coupled laser optics. The E axis for layer application uses similar dry run bearings and linear carriages to the X- and Y axes, and is driven by a stepper drive via two threaded shafts synchronised by a timing belt. It is oriented in parallel to the X axes, and uses an optical end-stop position sensor. The Z axis adapter carrying the build-platform can be moved up and downwards. It is driven by three fine-thread shafts also synchronised by a timing belt and driven by stepper drives. An optical position switch is used to get a fixed reference position.

### Laser system: source, optics, driver

In the same way, as for the rest of the device, the laser system must survive the various forces undergone during the flight and must be equipped with a cooling system independent of air convection. The laser system consists of a fibre-coupled diode laser as source, laser optics attached to the fibre end, diode driver electronics, and a laser cooler system. For the selective laser melting process we use a laser diode bar module, type Coherent IS58, with max. 283 W optical power delivered at centre wavelength of 976 nm into a 100 µm fibre core. The laser optic is assembled using standard C-mount lens adapters, a SMA-type fibre adapter, and plano-convex lenses. All 1”-lenses are *λ*/10 grade uncoated Fused Silica lenses (Edmund Optics, Inc.). The distance between the focal plane of the front lens to the build’s topmost layer is 35 mm, resulting in a minimum laser spot of Ø 80 µm. A temperature sensor is attached to the optics assembly in order to rapidly detect any overheating – mostly due to the presence of dust. The laser diode driver used is a MESSTEC type MSM 100-25. An interlock circuit and more crucial safety features are in place to prevent laser power emittance for example while the hatch is open, rendering the overall set-up a class 1 laser system. By default, the laser diode is to be cooled by cooling water at constant temperature and flow-rate. This of course can hardly be achieved in a compact sounding rocket payload. Therefore, an alternative cooling solution was built to purpose. The cooling loop uses a pump to circulate the coolant and transport heat into the purpose-built heat sink. The heat sink uses finned pipes to transfer thermal energy from cooling liquid to a phase change material, Rubitherm RT28 HC. This allows the laser to be used continuously at full power for a limited time of 10 min, which is more than sufficient for experiments conducted in limited microgravity time. For lab experiments, however, an external cooling system is used for prolonged constant laser operation.

### Pressure chamber and gas flow

The manufacturing process takes place in a closed, hermetically sealed environment inside an experiment chamber to ensure a defined atmosphere of constant pressure, and especially to guarantee an oxygen concentration during processing of ca. 0.4 %. The experiment chamber is designed to resist pressure differences between the inside and outside. Firstly because of the inner under pressure when evacuating the chamber: a difference of up to 1 bar is needed in preparation for flushing with the desired gas composition. Secondly because of the inner over-pressure when the outside pressure drops during space flights: resistance to a difference of up to 2 bar is required.

The building process happens in an insert: an easily removable cartridge (shown in Fig. 2) accessible through a hatch which includes the powder container, the Z- and E axis drives, and the build-platform. This way the build-platform or powder – even the whole cartridge – can easily be exchanged when the device is in flight configuration before lift-off. The powder must be stabilised against weightlessness when it is part of the powder bed and yet be flowable enough to apply new layers to this bed. To this end, within the cartridge, a closed-loop gas flow is used to force powder particles towards the build-platform, as done in the gas flow-assisted powder deposition previously described^24^. A rubber hose is attached to the mounting adapter for the build-platform, allowing vacuum generation below it. Because of the platform’s porosity, a gas stream perpendicular to the surface is generated, pushing powder particles towards it. Outside of the cartridge, the gas stream is directed through a set of 10 µm particle filters, and a mass flow sensor (Red-Y series, Vögtlin Instruments GmbH), into two parallel acting controllable diaphragm pumps, type 3111.610 (Boxer GmbH). While filters and flow sensors are placed outside the chamber, the pumps are inside the chamber, to avoid external pressure differences on the pumps. The pump outlet is left open, back into the chamber.

### Build-platform and layer application

The MAPHEUS sounding rocket offers 6.5 min of uninterrupted microgravity time, which is enough to serve as a proof of feasibility. Because of this time constraint, the build-platform need not be very large: it was chosen to measure 45 mm × 45 mm and is machined from a 5-mm-thick porous sinter body of stainless steel 1.4404, with pore size of 8 µm.

To apply a layer of feedstock to the build-platform, a control sequence is used as follows. First, the Z axis is lowered by the desired layer thickness, setting the build-platform into position to receive a fresh coating of powder. The Z axis is only homed before the first layer to find the reference position beforehand, and calibrated to the exact thickness of the build-platform. After lowering the build-platform in Z, the build-platform adapter is moved below the powder reservoir, along the E axis.

When initiating the movement, a piezo-actuator placed at the bottom of the powder reservoir is activated to produce harmonic oscillations of the compartment. The frequency is adjusted close to the Eigenfrequency of the mechanical system to maximise energy transferred. Through this, kinetic energy is introduced to the powder, unblocking possible particle clusters and increasing flowability. Additionally, the gas flow through the build-platform is active during the whole process, and now passes through the powder reservoir forcing particle transport towards the build-platform. A filter on the top of the reservoir allows gas intake from the chamber. A pause is programmed to give time for particle deposition, which usually requires a couple of seconds. Afterwards, the E axis moves the build-platform back to its original position under the laser optics. During this movement, particles exceeding the layer thickness are held back, producing a smooth layer with the programmed thickness. The rest of the feedstock remains in a completely sealed compartment. It is important to point out that in microgravity, the necessity of the gas flow means that any part built has to be a framework of single lines (curved or straight) to allow passage of the gas (see Fig. 7 and in Supplementary Fig. 2).

### Power management

The device can use external power when used in the lab but must contain its own power source when in the rocket. Therefore, it was designed to be supplied with electric energy via an umbilical connector from an external power supply when on Earth and by internal batteries when in flight. In off-state the payload can only be started by applying 16−20 V_dc_ on the umbilical connector – for safety reasons not from battery power. LiFePO_4_-type batteries^40^ (two packs in 6S3P configuration) can be installed or removed by one hatch each, and switched on and off individually. The nominal capacity of 13.8 Ah allows for 30 min of experiment time at full power intake.

### Process control

For the process to run smoothly and without being overdependent on user control, the actual manufacturing process is driven by a controller board with interfaces to control stepper drives, laser power, and power switching to read and write digital inputs as well as read and control temperature. We use a Smoothieboard 5XC V1.1 from the open hardware project Smoothieware.org. This board controls stepper drives for X-, Y-, Z-, and E axes, and respective end stops and position switches, through onboard drivers.

### Data acquisition and communication interface

Because there are many parameter changes (such as temperatures, gas flow velocity, axes positions) and operations happening simultaneously during the AM process, it is critical to have a clear and comprehensive communication interface to monitor the parameters as well as record the process to maintain an overview and be able to look back at the experiment and find potential sources of error. To monitor the manufacturing process and collect in-situ data, a data recorder system is used. It is a modified single-board computer, type Gigabyte GA-SBCAP3450, running a LabVIEW virtual instrument (National Instruments Corp.). The peripheral hardware used are: two cameras and two analogue and digital interface cards, respectively connected by USB. The following sensors are attached to the data recorder:

- High definition camera for overall view of the build-platform,
- Microscope for detailed view of the Z-movement,
- Recording batteries and external power source voltages,
- Pressure transducer to measure inside the experiment chamber, measuring over a range of 0–5 bar,
- Mass flow sensor for measurement of the gas stream through build-platform, range 0.3–15 NL min^-1^,
- Pt1000 temperature sensors, i.e., attached to laser diode, laser optics, and build-platform,
- Tilt sensor reads within a range of ±1 g along the *Z* axis, and can be used for microgravity detection,
- O_2_-sensor Roßmann Electronic O2S-FR-T2 to measure inside the experiment chamber, range 0.1–25% O_2_,
- Digital inputs read flight events for lift-off and begin or end of microgravity, as reported by MAPHEUS service system.

All sensor data, machine states, telemetry and telecommand data, as well as potential errors are logged into an onboard file, usually at a rate of 10 Hz which – if necessary – could easily be increased to 100 Hz and above. Video recording is at 30 fps. The data recorder sends a telemetry data stream and a TV signal and receives telecommand packages. A bi-directional RS485 serial communication to the MAPHEUS Service Module is used, according to a predefined interface description. The transmitted TV signal displays live views of camera and microscope, and additionally displays a selection of the most important sensor data and system health information. Telecommand packages received from the ground station are checked, interpreted, appended to a logfile, and executed immediately.

### Ground station and ground support equipment

**Fig. 3:**
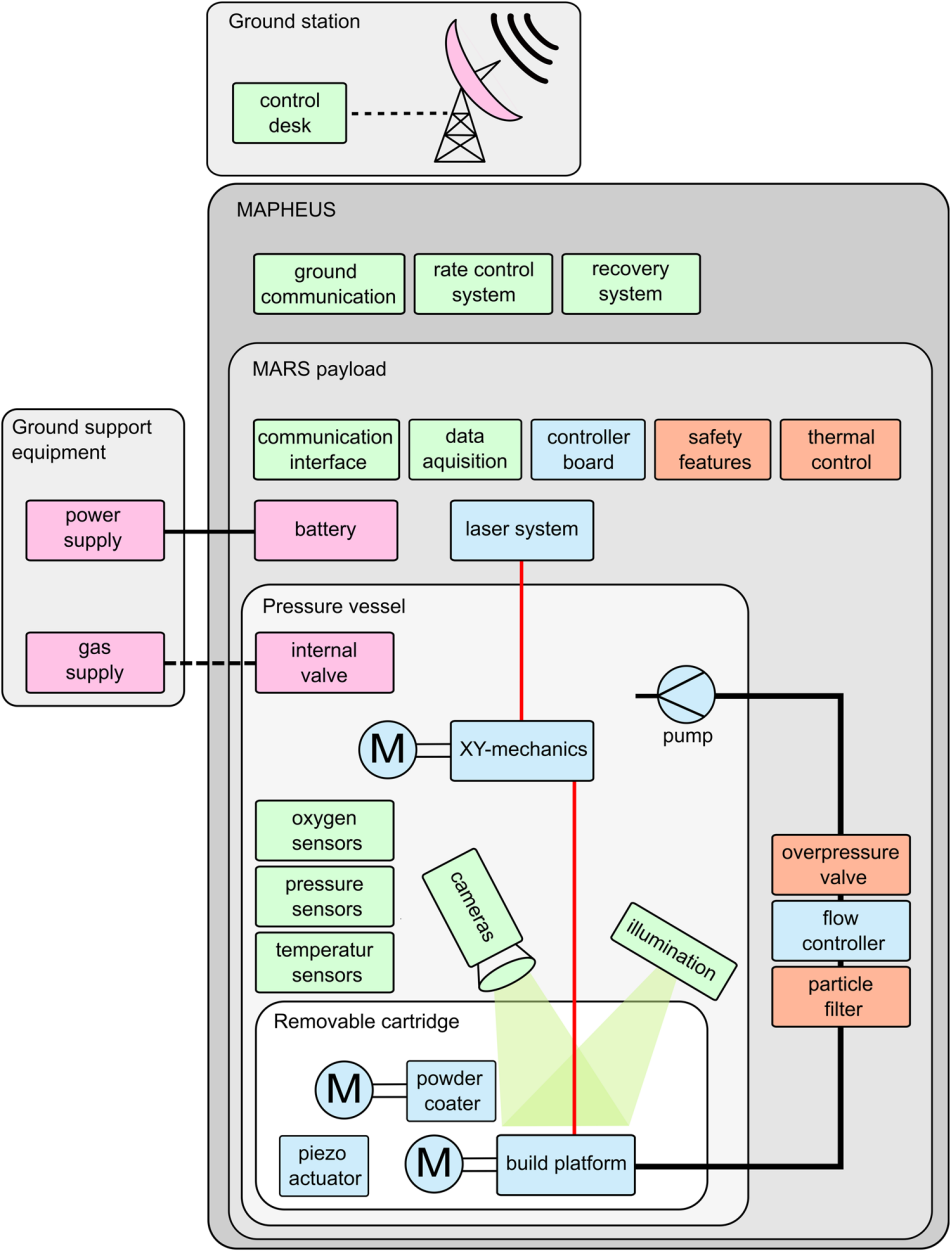
System diagram of hardware and communications of MARS-M within MAPHEUS. This diagram deconstructs the system into subgroups starting from the lowest level of the removable cartridge, up to the MAPHEUS payload support systems and vehicle.

Though the device is meant to function without assistance or additional equipment during the flight, some large equipment is necessary to prepare before launch. As such, this ground support equipment (GSE) is usually not integrated into the payload to save weight. Such systems are placed in a protective box close to or on the launcher. In this case, the GSE contains: a power supply, an Ethernet interface, a vacuum pump, valves a gas bottle, and a pressure transducer. The power supply is connected to the payload’s umbilical and before lift-off, the supply is switched to internal batteries. The ground station used for MARS-M is a desktop computer, running a LabVIEW virtual instrument. It receives telemetry data and TV downlink, and sends telecommand data packages according to user input. Data, flight events, and videos are displayed for operators and logged into files. Additionally, it is connected to the GSE by Ethernet. Until lift-off, this Ethernet connection reaches the Smoothieboard, data recorder, and Ethernet interface inside the payload via an umbilical connector. This data connection can also be used to operate the experiment in lab settings and enables remote access to the data recorder and controller board to transfer log files or run maintenance work. The way the manufacturing process is linked to MAPHEUS payload support systems and the ground station is represented in Fig. 3.

### Programming and machine limits

Setting-up the previously introduced hardware and software is the first step to start building parts. The next step is to adapt the manufacturing process to the chosen material. Whereas the software and configurations are dependent only on the device used to build parts, the combination of laser power, scanning speed, layer thickness, and scanning strategy (and sometimes gas composition^41^) is dependent on the selected powder. To adjust these parameters, MARS-M uses standard G-code, Smoothie flavour, and additionally a set of M-commands specific to this machine. Most common G- and M-commands used in MARS-M are in the firmware documentation (https://smoothieware.org/supported-g-codes, last consulted 28.02.2023), together with the available arguments and valid variable ranges, as well as coding examples. Each argument of a G-command is a decimal string with up to four digits of precision, while for an M-command the decimal string represents an integer value. Codes may be run from internal memory card (necessary during flight), sent via a telnet connection from the ground station, or individual commands can be given by using the web interface.

When building with a different powder composition, the laser speed, scanning velocity, hatching distance, and layer thickness have to be adapted and optimised. In order to generate G-codes to build test bodies, we use software scripts to output the code files (see next section). Meanwhile, to generate more complex 3D geometry, CAD models can be processed through a powerful and approved slicer, such as open source software Cura or Slic3r to generate a G-code intended for FFF-type printers, with properly adjusted settings for this machine. It is then necessary to post-process this G-code with LabVIEW software created for the purpose of sorting out FFF-specific commands and arguments while maintaining the geometry information, adding commands and arguments for laser process and gas flow control as well as the G-code sequence for powder application, and making sure that no command violates machine limits, such as the maximum axis ranges.

## Results and discussion

### Flight qualification and calibrating experiments

Prior to its participation in sounding rocket flights the complete MARS-M payload was first tested in microgravity on parabolic flights with a Novespace Zero-G aircraft, using 62 parabolas on two flight days with about 20 s of microgravity per parabola. In order to meet safety requirements for parabolic flight, we slightly adapted the hardware without impact on the function. While the powder handling mechanics and control algorithm were calibrated and adjusted to weightlessness during the parabola, no laser melting was performed due to short microgravity time. The stack of powder layers was analysed after each flight. The gas flow settings that proved suitable for microgravity during parabolic flights were also applied to lab experiments and MAPHEUS flights. With these settings, applying one layer of powder takes 15 s. For the parabolic flights, powders from stainless steel 1.4404 of particle size 20−53 µm and Zr-based metallic glass AMZ4 (now AMLOY-Zr01, industrial grade, Heraeus AMLOY) at particle size 45−100 µm were used. After qualifying for parabolic flights, the device was then qualified for operation under vacuum conditions during environmental testing. In this configuration the laser system was successfully qualified for spaceflight on MAPHEUS.

**Fig. 4:**
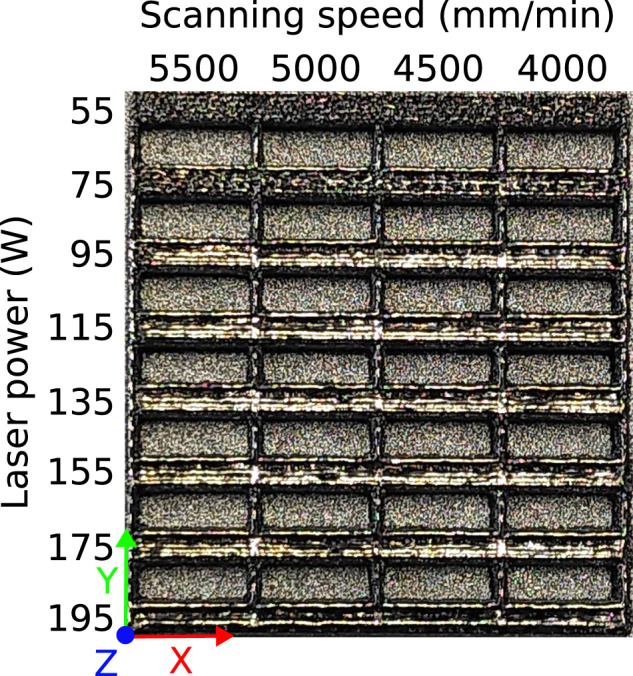
Example of segments of AMZ4 with varying parameters. Differences in parameters lead to optical differences in surface smoothness and shine. Where the laser power is lowest, so is the surface smoothness. Where it is highest, it becomes harder to distinguish the individual lines building up the segments. The overall size is ca. 26 mm × 28 mm.

Optimisation of the manufacturing process itself was performed on-ground by building elements of increasing dimensions: lines (1D), thicker segments (1.5D) up to 2 mm thick (see Fig. 4), surfaces (2D) up to 2 mm thick, and objects (3D, each layer potentially different, see Supplementary Figs. 2 and 3). Varying ranges of parameter values for laser speed (1000−5500 mm min^−1^) and laser power (55−230 W) were used to build, inspired by the literature^42–44^. A 2D grid of segments is created on the build-platform with increasing laser power along the Y axis, and increasing laser scanning speed along the X axis, as shown in Fig. 4.

At the beginning of the optimisation process, a wide range of laser power and scanning speed is used. Typically, the segments at the extremities of the first test-build are visibly flawed and can be excluded in the next build: high porosity and balling at too high energy input (higher power and lower speed, see bottom-right corner of Fig. 4), or very rough surface and visible presence of incompletely melted powder at insufficient energy input (lower power and higher speed, see top-left corner of Fig. 4). In the latter case, the energy density can be so low that though it fuses powder particles together it is not sufficient to weld them to the build-platform. An additional impact of several parameters and their evolution can be seen in Supplementary Fig. 1. By narrowing down the range of parameters for each new set of segments, one can rapidly find settings deemed “optimal” within the constraints of the device: the powder is fully melted with the lowest balling, least porosity, smoothest and shiniest surface. Additionally, in the case of a metallic glass the crystalline fraction should be as small as possible – ideally non-existent.

**Fig. 5:**
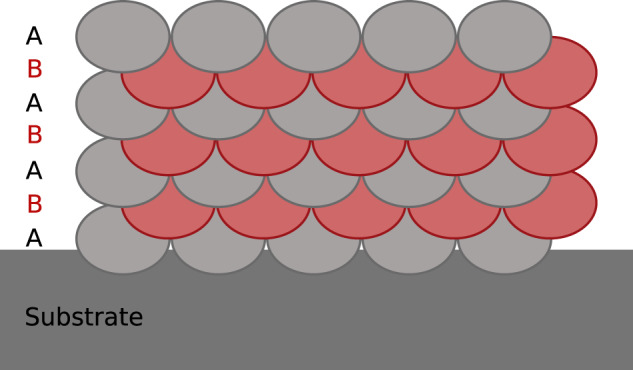
Schematic representation of the segments “A + B” scanning strategy. The laser moves in the direction perpendicular to the figure, and each layer is displaced by half a hatch distance to the previous one.

The quality of the various resulting builds is first analysed through optical examination (surface aspects). When the parameters are narrowed down further, scanning electron microscopy (SEM, Zeiss Merlin) and x-ray diffraction (Bruker, D8Advance) become necessary to observe differences in microstructural aspect: homogeneity, smaller sized porosity, crystalline fraction. This method led to the selection of the scanning strategy “A + B” shown in Fig. 5. This strategy involves two different layers A and B, which are almost identical but displaced by half a hatch-distance as well as reversed in direction of the laser, which moves along the axes perpendicular to the represented plane (A: towards the reader, B: away from them). This strategy was found to reduce porosity and holes when building a dense part.

**Fig. 6:**
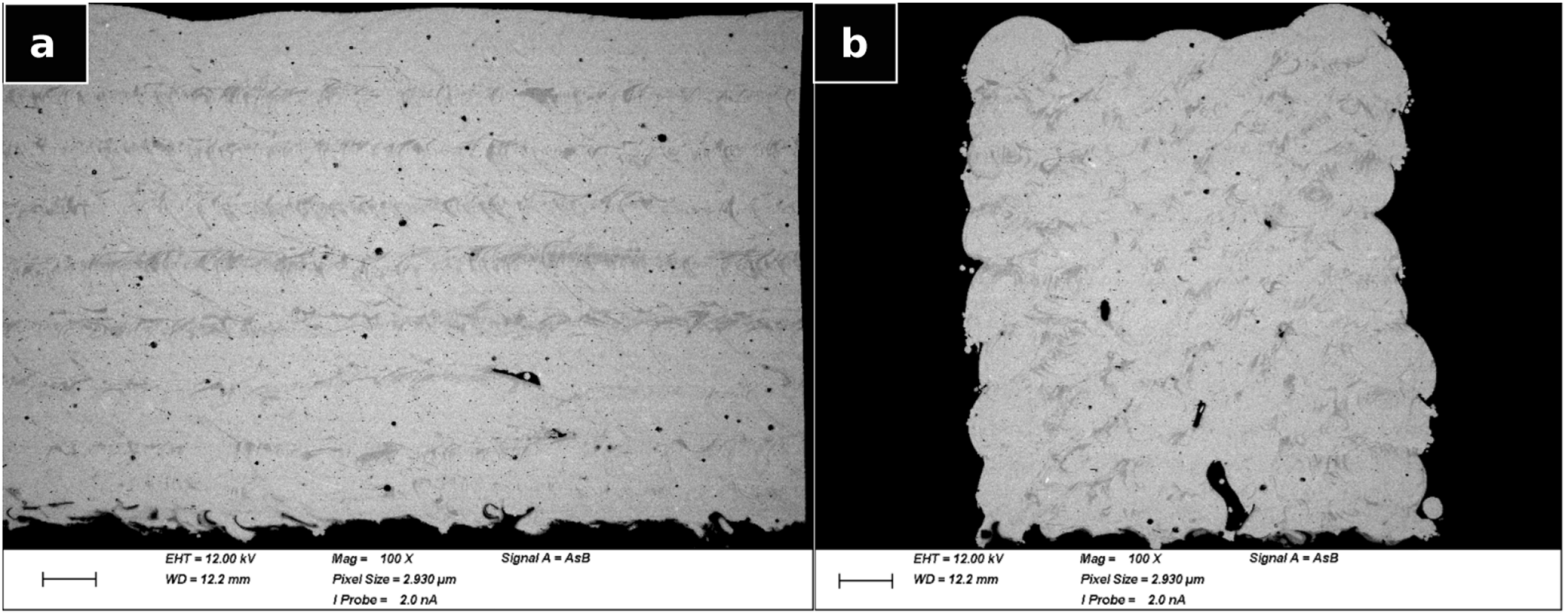
SEM pictures. SEM of cross-sections of AMZ4 built with 115 W at 5500 mm min^−1^, parallel (**a**) and perpendicular to laser movement (**b**). Scale is 200 µm.

In the case of a metallic glass, once the part density is satisfactory a closer examination of the crystalline content is performed. Different laser powers and scanning speeds produced different crystalline fractions. The results of SEM (at 12 kV accelerating voltage using a backscattered electron detector and a working distance of 12.2 mm) of the microstructure of a part built from AMZ4 with 115 W at 5500 mm min^−1^ with the “A + B” strategy over 20 layers of 100 µm each is shown in Fig. 6. Though all segment samples presented some crystalline content, in this figure a part with higher crystalline content was chosen and the contrast was increased for visibility of any difference in microstructure. The part displays crystallised regions (dark areas in Fig. 6) in the inter-boundary regions. In Fig. 6, these regions appear periodically in the left image, whereas their presence in the right image seems more chaotic until one compares it with Fig. 5. It is especially clear near the edges of the part that the crystallised zones are at the boundaries between slugs.

**Fig. 7:**
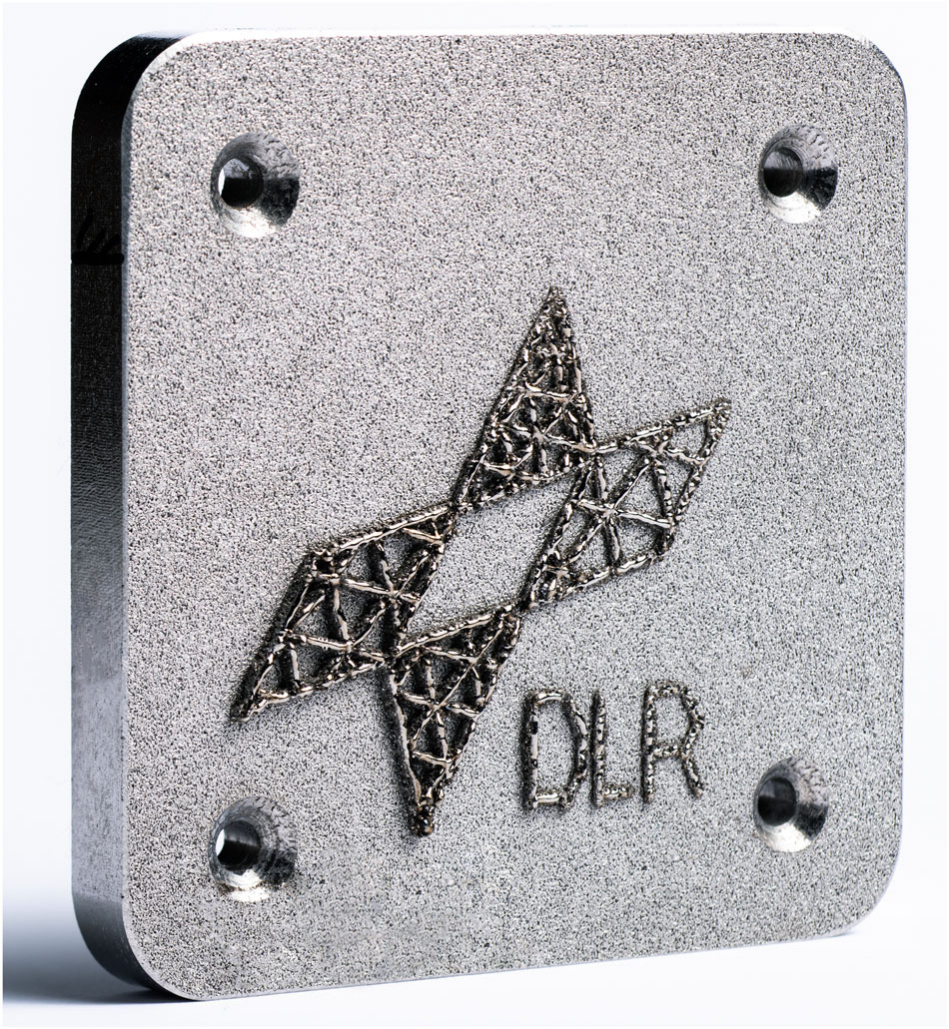
Part built from eight layers from AMZ4 powder in microgravity. The part is completely welded to the platform, no detaching is observed. The surface of the build is mostly smooth and has a silvery shine.

The parameters were optimised in the previously described manner for various materials: AMZ4, Vit105, Ti6Al4V, and steel 316 L. For the glassy compositions, the laser power and scanning speed producing the smallest crystalline fraction were selected. The parameters determined for AMZ4 in the lab (laser power 75 W, scanning speed 4000 mm min^−1^) were then used for the builds during the rocket flight campaigns (see Fig. 7).

### Characterisation of a Zr-based metallic glass microstructure built using MARS-M

Space flights with MARS-M have since been conducted in May 2021 onboard MAPHEUS-11, in December 2021 on MAPHEUS-10, and in January 2022 on MAPHEUS-09. AMZ4 powder (industrial grade, Heraeus GmbH) with particle size 15−45 µm was the feedstock on each flight, see properties in Table 1. In the following, we present a first analysis of the flight sample. In addition to the sample successfully built during the MAPHEUS-10 flight in microgravity, the same build was produced in lab conditions. The thickness of the flight sample is limited by the microgravity time (i.e., 6.5 min) as visible in Fig. 7, so that it can not be removed from the build-platform without being destroyed. Therefore, the synchrotron measurements were run in transmission with the beam perpendicular to the build-platform which was thinned down to around 500 µm to limit the signal from steel without risking destroying the flight sample.

**Table 1:** Properties of AMZ4 powder (industrial grade, Heraeus GmbH) with particle size $15-45$ µm.

Property	Value
Composition (at-%)	Zr_59.3_Cu_28.8_Al_10.4_Nb_1.5_
Microstructure	amorphous
Liquidus (°C)	920
Solidus (°C)	870
Glass transition (°C)	400
Crystallisation (°C)	475

**Fig. 8:**
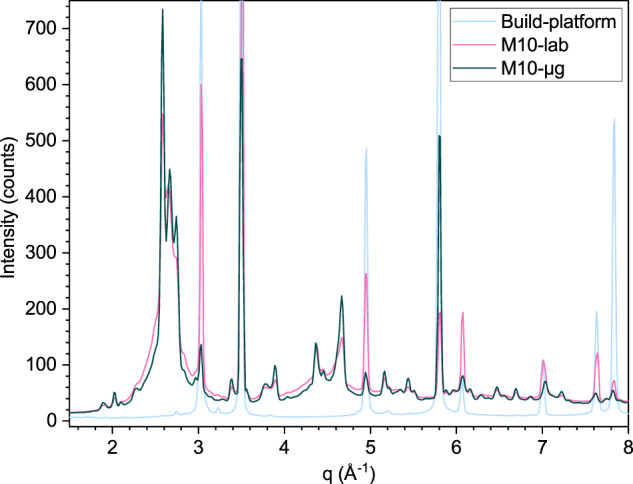
M10-µg sample vs. M10-lab sample in synchrotron. A comparison of synchrotron diffraction (in transmission) patterns of the MAPHEUS-10 flight sample (M10-µg), of the equivalent lab-sample (M10-lab), and of the steel build-platform. This last pattern helps to discriminate the Bragg peaks from steel present in the other patterns and the peaks confirming that neither M10-µg nor M10-lab are fully amorphous. The platform pattern intensity has been scaled for clarity.

Some results of the comparison between the flight sample (called M10-µg, built with laser power 75 W, scanning speed 4000 mm min^−1^) and the lab sample (M10-lab, same parameters) are presented in Fig. 8 as two typical curves integrated from the diffraction patterns. The signal from the build-platform is shown for peak recognition. The steel from the build-platform is readily noticeable and the samples are partly crystalline. The differences between M10-µg and M10-lab are slight and could in part be linked to a slight difference in thickness as M10-lab is two layers (i. e., 200 µm) thicker.

**Fig. 9:**
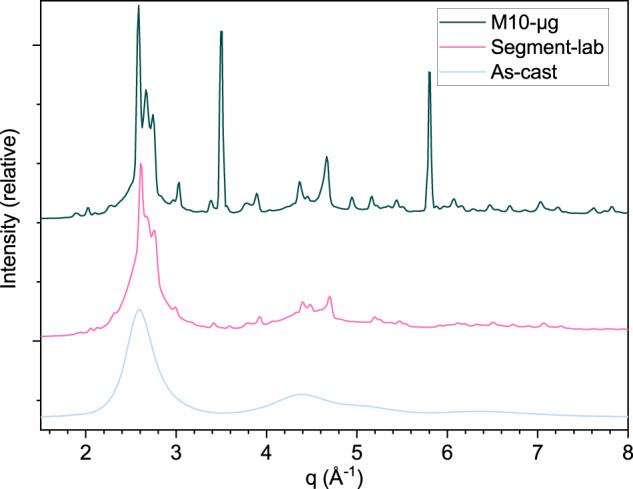
M10-µg sample vs. segment-lab sample and cast sample in synchrotron. A comparison of synchrotron diffraction (in transmission) patterns of the MAPHEUS-10 flight sample (M10-µg) and the ground sample (segment-lab) built with the same parameters: 75 W, 4000 mm min^−1^. The segment was large enough to be removed from the build-platform and as such does not present the Bragg peaks from steel which are still present in the M10-µg pattern. A pattern from a cast amorphous AMZ4 sample is also shown for comparison. It is clear that the segment-lab is partly crystalline.

The segment of AMZ4 built in the style of Fig. 4 with the same parameters as the M10 samples has a lower crystalline fraction as shown in Fig. 9 where it is compared to M10-µg. The segment was large enough that it could be mechanically removed from the build-platform and therefore does not present Braggs peaks from steel.

As explained above, MARS-M stabilises powder in microgravity using gas streaming through a porous build-platform. The gas flow and pores conceivably affect the melt flow dynamics as well as the heat transfer, but the extent of this influence is so far undetermined.

To conclude, the samples of AMZ4 were not completely amorphous. For AMZ4 this can be at least partly explained by the fact that in industrial PBF-LB processes, scanning speeds usually are much higher than is achievable using MARS-M, which will result in higher portions of amorphous material in the end-product. This is due to different design criteria and optimisation goals. While in industrial processes speed is a highly weighted factor, for rocket payloads compactness and robustness are essential. Nevertheless, ground experiments with a higher number of layers show that a large portion of the sample is amorphous. The crystalline regions are located in the interlayer regions, which requires further investigation. To improve our process further, different build strategies are being developed to reduce internal stresses, and different materials are being tested.

However, samples of metallic glass were successfully built from AMZ4 powder onboard a sounding rocket. This extends the validity of the gas flow concept for powder handling in microgravity. The next step is to run the process on an orbital platform to have more microgravity time available.

### Supplementary information

Supplementary Information

## Data Availability

The datasets used and/or analysed during the current study are available from the corresponding author on reasonable request.
