# Supplementary material for: Additive manufacturing of metallic glass from powder in space
Source: NPJ Microgravity. 2023 Oct 6;9:80. doi: 10.1038/s41526-023-00327-7 (PMC10558431; doi:10.1038/s41526-023-00327-7)
Supplement: Supplementary file 1 — Supplementary Information [file 41526_2023_327_MOESM1_ESM.pdf]

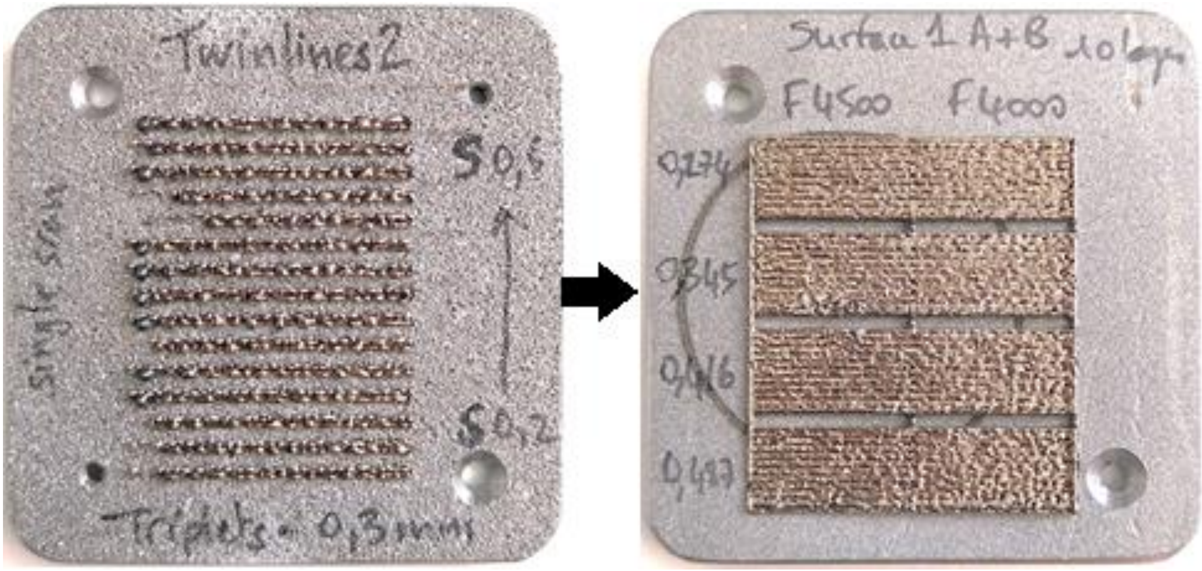

Supplementary Figure 1 - Example of progress made in the manufacturing of AMZ4 from two lines side-by-side (one layer) to surfaces (ten layers), with different parameters. On the left, balling is clearly visible especially where the laser starts its course on the left of each line, while some sections were insufficiently welded to the platform.
On the right, no detaching is seen and the surface is considerably smoother. Between the left and the right sample, the layer thickness was reduced; the build-platform porosity was changed to finer pores; the powder grain size was changed from 45−100 µm to 15−45 µm; vertical lines were also added at extremities (in case of detaching) and at parameter changes along X (for clarity).

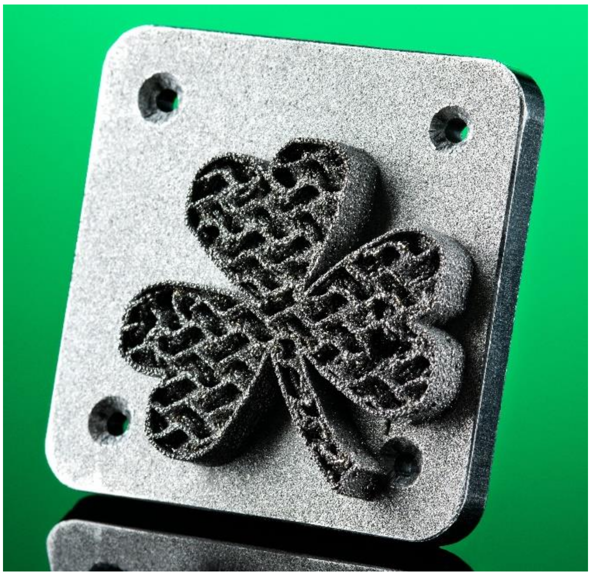

Supplementary Figure 2 - Example print 1. Shamrock built with Ti-6Al-4V with gyroidal infill.

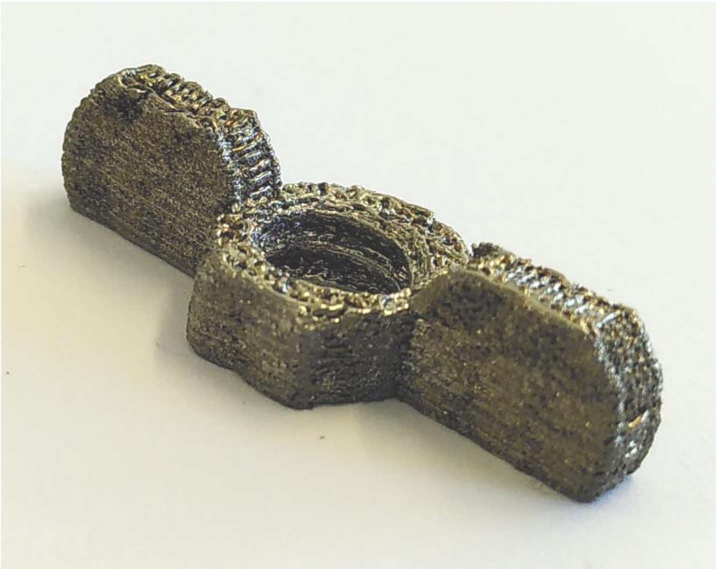

Supplementary Figure 3 - Example print 2. Wingnut built with AMZ4 powder, rectangular mesh infill.
